# Acylated Ghrelin and The Regulation of Lipid Metabolism in The Intestine

**DOI:** 10.1038/s41598-019-54265-0

**Published:** 2019-11-29

**Authors:** N. Auclair, N. Patey, L. Melbouci, Y. Ou, L. Magri-Tomaz, A. Sané, C. Garofalo, E. Levy, D. H. St-Pierre

**Affiliations:** 1 CHU Sainte-Justine Research Center, University of Montreal, Montreal, H3T 1C5 Quebec Canada; 2 Department of Nutrition, University of Montreal, Montreal, H3T 1A8 Quebec Canada; 3 Department of Exercise Science, University of Quebec in Montreal (UQAM), Montreal, H2X 1Y4 Quebec Canada; 4 Institute of Nutrition and Functional Foods (INAF), Laval University, Quebec, G1V 0A6 Quebec Canada

**Keywords:** Dyslipidaemias, Gastrointestinal hormones

## Abstract

Acylated ghrelin (AG) is a gastrointestinal (GI) peptide mainly secreted by the stomach that promotes cytosolic lipid droplets (CLD) hypertrophy in adipose tissues and liver. However, the role of AG in the regulation of lipid metabolism in the intestine remains unexplored. This study aimed at determining whether AG influences CLD production and chylomicron (CM) secretion in the intestine. The effects of AG and oleic acid on CLD accumulation and CM secretion were first investigated in cultured Caco-2/15 enterocytes. Intestinal lipid metabolism was also studied in Syrian Golden Hamsters submitted to conventional (CD) or Western (WD) diets for 8 weeks and continuously administered with AG or physiological saline for the ultimate 2 weeks. In cultured Caco-2/15 enterocytes, CLD accumulation influenced CM secretion while AG reduced fatty acid uptake. In WD hamsters, continuous AG treatment amplified chylomicron output while reducing postprandial CLD accumulation in the intestine. The present study supports the intimate relationship between CLD accumulation and CM secretion in the intestine and it underlines the importance of further characterizing the mechanisms through which AG exerts its effects on lipid metabolism in the intestine.

## Introduction

The alarming increase in the incidence of obesity is a major issue for social health authorities worldwide. Among the various metabolic complications of obesity, lipid and lipoprotein disorders are prevalent and predispose to cardiovascular diseases. In particular, postprandial dyslipidemia constitutes a significant risk factor for the development of atherosclerosis, ischemic stroke and other chronic diseases^1^. Although insulin resistance and amplified intestinal chylomicron (CM) production are considered important processes for the increased postprandial lipemia^2,3^, additional studies are needed to unravel the underlying mechanisms. This is mostly important since an increasing proportion of individuals frequently consume lipid-enriched meals throughout the day and therefore spend most of their time in the postprandial state.

As recently reviewed by our group^4^, apolipoprotein B-48 (apoB-48)-containing lipoproteins are formed in the endoplasmic reticulum (ER), moved to the Golgi apparatus to complete their maturation and are secreted as chylomicrons (CM) across the basolateral membrane of enterocytes in the postprandial state. Once CM particles reach the circulation, their triacylglycerol (TAG) content is hydrolyzed by lipoprotein lipase (LPL) to provide peripheral tissues with non-esterified fatty acids (NEFA). This process reduces TAG content of CM to produce CM remnants (CMR). Thereafter, CMR particles are internalized by the liver or accumulated in the arterial endothelium following chronic postprandial dyslipidemia, thereby increasing the risk of developing cardiovascular diseases. Considerable efforts are currently directed at discovering the factors, which contribute to the pathophysiological processes leading to postprandial dyslipidemia. The rate at which CM are processed is intimately correlated with the accumulation of cytosolic lipid droplets (CLD)^4^, which contain a hydrophobic core of TAG and cholesteryl ester surrounded by a phospholipid monolayer comprising few free cholesterol molecules. CLD are formed by the clustering of neutral lipids within the hydrophobic region of the ER^4^. CLD accumulation and hypertrophy occur in organs and tissues with key metabolic functions such as adipose tissues and liver^5^. In the latter, excessive CLD accumulation is intimately associated with the development of non-alcoholic fatty liver disease (NAFLD)^6^. As recently reviewed by our group, CLDs are key lipid reserves that undergo hydrolysis/re-esterification to provide the ER with sufficient TAG for CM assembly and secretion^4^. In the intestine, CLDs are suggested to exert their beneficial effects by delaying the transfer of dietary TAG to the ER and CM synthesis/secretion while optimizing the system’s capacity to cope with the postprandial lipid surge^7^. However, important questions remain regarding the mechanisms regulating CLD accumulation and their functional outcomes on lipid metabolism in the intestine.

More recently, CM production has been shown to be regulated by gut-derived peptides, which are secreted by enteroendocrine cells located throughout tissues of the stomach, small intestine and colon^8^. Many of these hormones are key neuroendocrine regulators of vital functions such as eating behaviours, glucose homeostasis, energy expenditure and lipid metabolism. Some of these peptides such as glucagon-like peptide 1 (GLP-1), glucagon-like peptide 2 (GLP-2), neurotensin and leptin were previously shown to control CLD accumulation and/or CM secretion in the small intestine^9–11^. Surprisingly, little information is available for ghrelin, an orexigenic GI peptide mainly secreted by X/A cells of the stomach but also in decreasing concentrations through the distal part of the intestine^12^. In the circulation, ghrelin can be found under the acylated (AG) and unacylated (UAG) forms^12^. The growth hormone secretagogue receptor of type 1a (GHSR-1a) is recognized as the endogenous receptor mediating the activity of AG in the periphery and in the central nervous system (CNS)^12,13^. Moreover, AG can stimulate food intake by activating NPY and agouti-related peptide (AGRP) neurons of the hypothalamus^14^. In the periphery, ghrelin promotes CLD accumulation in adipose tissues and liver^15,16^. Both UAG and AG are also reported to influence GI motility. Nevertheless, important questions persist regarding their effects on lipid metabolism and/or CLD accumulation in the intestine^17–20^. Apart from its stimulatory effect on gut motility, there is scarce information regarding the role of ghrelin in the digestive tract, therefore, it is critical to determine the impact of this hormone on lipid metabolism in the small intestine. To do so, this study intended to clarify the role of AG on CLD accumulation and CM formation using Caco-2/15 enterocytes as well as in the intestine of Syrian Golden Hamsters, two unique and representative models of human lipoprotein physiology. In the conditions tested, AG did not influence CLD accumulation or CM secretion in Caco-2/15 enterocytes. However, in Syrian Golden Hamsters, AG potently reduced postprandial CLD accruement while enhancing CM output and promoting systemic dyslipidemia.

## Results

### Oleic acid treatment and CLD accumulation in Caco-2/15 cells

**Figure 1:**
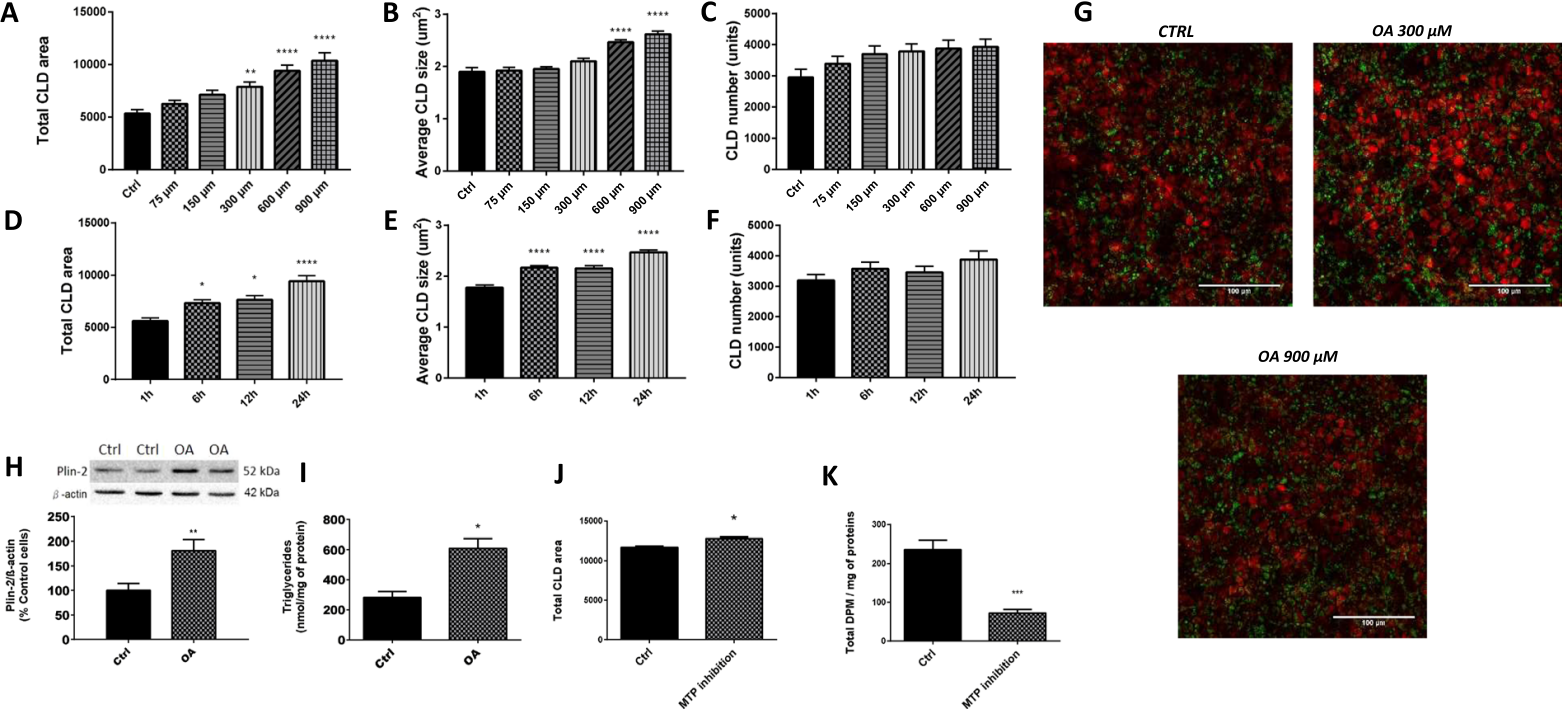
CLD characteristics in response to treatment with oleic acid (OA) in Caco-2/15 cells. Caco-2/15 cells were treated with 0, 75, 150, 300, 600 or 900 µM of BSA-conjugated oleic acid (OA) for 24 h (**A**–**C**). Enterocytes were also incubated with BSA-conjugated oleic acid (600 µM) for 1, 6, 12 and 24 h (**D**–**F**). Then, cells were fixed while both their nuclei (red, DAPI) and CLDs (LipidTox, green) were stained. CLD total area, average size and numbers were determined. Examples of the pictures obtained are shown at three different concentrations (**G**). Caco-2/15 cells were incubated 24 h with or without BSA-conjugated OA (600 µM) while Plin-2 (**H**) and triglycerides (**I**) contents were measured. Plin-2 was determined by Western blots and results are illustrated as % of control cells after the data were calculated as densitometric ratios of Plin-2 to β‐actin. A representative blot is shown, illustrating an experiment in duplicate on the same gel and at the same time exposure. Caco-215 cells were incubated 24 h with or without OA (600μM) and with a MTP inhibitor (0.1 µM, Lomitapide Mesylate, BM-201038) on their apical side. Thereafter, cells were incubated (24 h) on their apical side with radiolabeled oleic acid with or without Lomitapide Mesylate (0.1 µM). Cytosolic lipid droplets were analyzed (**J**) while chylomicron secretion was measured by analysis of the quantity of ^14^C found in the CMs fraction in the basolateral media. (**K**) Results are shown as mean ± SEM (n = 3 individual experiments); ^*^P < 0.05; ^**^P < 0.01; ^****^P < 0.0001 *vs* control treatment.

This section was carried out to determine the conditions promoting optimal CLD growth in cultured enterocytes. In Caco-2/15 cells incubated with OA, CLD expansion was stimulated from 1 h to 6 h while reaching its peak at 24 h (Fig. 1D,E). Also, in response to OA, total CLD area was increased in a dose-response manner (Fig. 1A) while maximal average CLD size was reached at concentrations of 600 and 900 µM (Fig. 1B). For both experiments, CLD numbers were not influenced by the incubation time or OA concentrations (Fig. 1C,F). These results were analyzed from images obtained by confocal microscopy and examples are shown in Fig. 1G. As illustrated, the optimal OA concentration/incubation time enhanced total CLD area by a factor of 2X when compared to control treatments. OA (600 μM for 24 h) also significantly increased Plin-2 (Fig. 1H) and TAG (Fig. 1I) levels by a factor of 2X in Caco-2/15 enterocytes.

### Inhibition of chylomicron secretion and CLD growth in Caco-2/15 cells

To examine the interaction between CM output and CLD accumulation, we used Lomitapide Mesylate, an inhibitor of microsomal triglycerides transfer protein (MTP), which blocks CM formation. Importantly, the presence of the MTP inhibitor did not alter cell viability and functionality since its addition to Caco-2/15 cells did not affect viable cell count (according to Trypan Blue Dye Exclusion Assay), transepithelial electrical resistance (TEER), sucrase and villin as biomarkers (results not shown). Lomitapide Mesylate significantly reduced CM secretion by 69% (Fig. 1K) while increasing total CLD area per microscopic field (Fig. 1J).

### Acylated ghrelin and fatty acid uptake by Caco-2/15 cells

**Figure 2:**
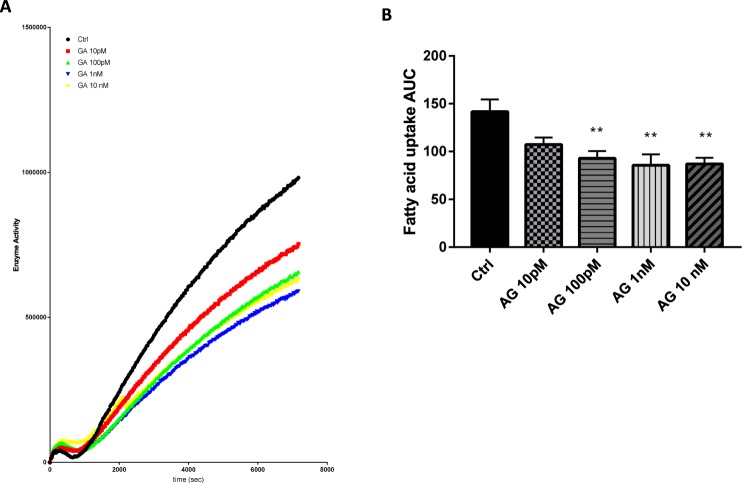
Uptake of fluorescent-tagged fatty acid derivatives inhibited by AG treatment. Caco-2/15 cells were pre-incubated with EMEM medium without FBS for 2 h and then treated with acylated ghrelin (AG) at 0 pM, 10 pM, 100 pM, 1 nM and 10 nM. Fatty acid (FA) uptake was measured every 30 seconds for 2 h (**A**) and the area under the curve (AUC) was calculated. (**B**) Results are shown as mean ± SEM (n = 3 individual experiments); ^**^P < 0.01 *vs* control treatment.

Caco-2/15 cells were incubated with different concentrations of AG (10 pM, 100 pM, 1 nM, 10 nM). AG significantly reduced FA uptake (kinetics and area under the curve, Fig. 2A,B) at all concentrations tested (P < 0.01 *vs* control treatment) but not at 10 pM. Interestingly, a plateau effect was reached in response to AG at concentrations above 100 pM.

### Acylated ghrelin and lipoprotein formation in enterocytes

**Figure 3:**
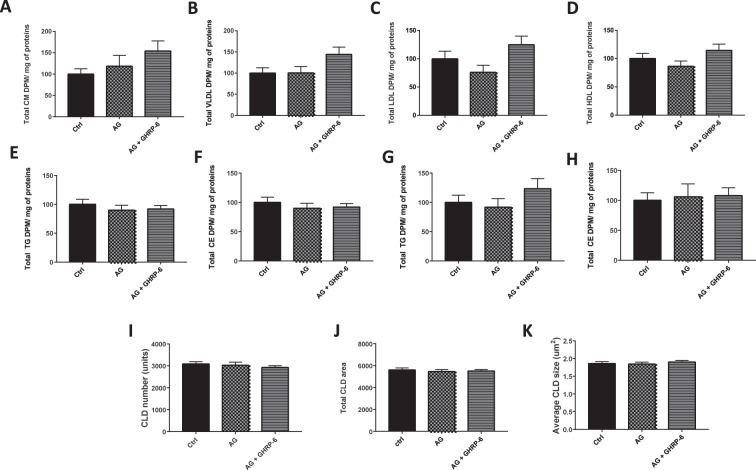
Acylated ghrelin effects on lipoprotein secretion, intracellular lipid metabolism and CLD accumulation in caco-2/15 cells. Caco-2/15 cells were incubated with radiolabeled [^14^C] on their apical side. Co-treatment with AG (100 pM) was carried out on the basolateral and apical sides for 24 h with or without a GHSR-1a antagonist, [D-Lys^3^]-GHRP-6 (50 µM). Relative quantification of non-VLDL/LDL/HDL (**A**), VLDL (**B**), LDL (**C**) and HDL (**D**) lipoproteins. Levels of apical TAG (**E**) and (**F**) cholesteryl ester (CE) as well as basolateral levels of TAG (**G**) and CE (**H**) were measured in enterocytes. After the incubation, cells were fixed and fluorescent markers for nuclei (DAPI) and neutral lipids (LipidTox) were used for CLD characterization using confocal microscopy. CLD total area (**I**), numbers (**J**) and average size (**K**) were measured. Results are shown as mean ± SEM (n = 2 experiments in in triplicate).

In response to AG (100 pM) with or without a GHSR-1a antagonist, [D-Lys^3^]-GHRP-6 (50 μM) on the apical and basolateral side, no modulation of lipoproteins, TAG or cholesteryl ester (CE) levels was noted in Caco-2/15 cells (Fig. 3). Similarly, no differences were detected in total area and number of CLD per microscopic field (Fig. 3).

### Influence of acylated ghrelin on syrian golden hamsters

**Figure 4:**
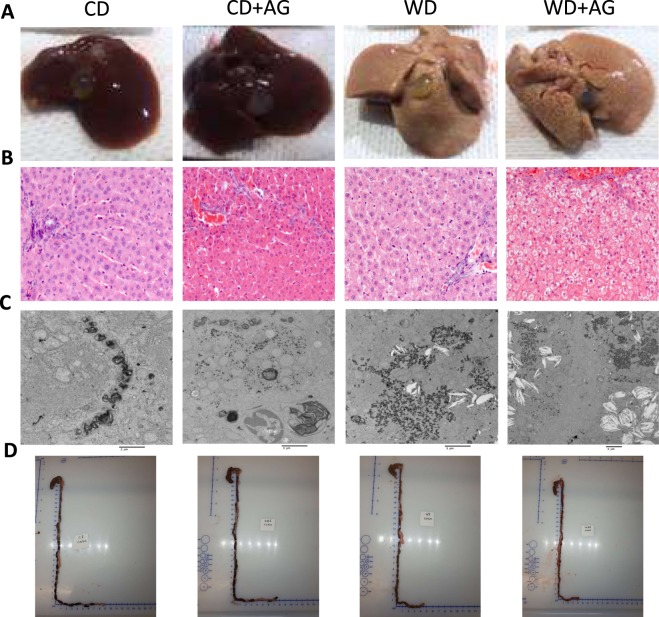
Effects of the Western diet on liver histology and the visual aspect of the colon. Syrian Golden Hamsters were submitted to conventional chow (CD) or Western (WD) diets for 8 weeks. For the last 2 weeks of treatment, animals were continuously administered with acylated ghrelin (AG, 100 nmol/kg/day) or physiological saline. Before sacrifice, hamsters were fasted for 16 h before receiving an olive oil gavage (200 µL). This figure provides a representative visual aspect of livers (**A**), their histology with hematoxylin-eosin staining (**B**) and their picture taken by electronic microscopy (**C**) for each treatment group. Also, a representative visual aspect of the colon of each experimental group is provided (**D**).

Since results obtained in cultured cell lines are not always representative of physiological conditions in living organisms, a Syrian Golden Hamster model was subsequently used to test AG’s effects on lipid metabolism in the intestine. As presented in Table 1, when compared with CD, WD significantly increased fasting plasma TAG, total cholesterol, free cholesterol, cholesteryl ester and non-HDL cholesterol concentrations, while having no significant effect on HDL cholesterol. This indicated that WD induced hyperlipidemia. With respect to CD treatment, WD rats also displayed increased liver weight. This observation is supported by an elevation of hepatic TAG and cholesterol content, along with an alteration in the visual aspect of their livers (Fig. 4A)*.* Importantly, the quantity of TAG doubled, but the level of cholesterol increased by 6 times more in the liver under the influence of WD. Accordingly, liver histology demonstrated a marked accumulation of cholesterol crystals along with the development of a micro and macro steatosis, and an abnormal height of the hepatocytes in WD-fed animals (Fig. 4B,C). On the other hand, the small intestine was characterized by a significant rise in weight and cholesterol content (Table 1). A reduction in colon weight was also observed in the WD group (Table 1) and this was also underpinned by the decrease of its overall length (Fig. 4D). Only a non-significant increase in glucose, insulin and HOMA-IR^2^ was observed without an increment in body weight and epididymal fat (Table 1). As for AG, it significantly increased body weight in treated hamsters under the two diets (Table 1). However, no significant difference in the caloric intake was detected between hamsters submitted to the different treatments. Nevertheless, WD + AG animals displayed significantly increased liver and epididymal fat pad weights compared to WD hamsters. AG infusions further increased TAG and total cholesterol levels in the plasma of WD animals, which therefore amplified systemic dyslipidemia. Finally, insulin resistance was slightly increased by AG under CD and WD, but the differences relative to fasting glucose, insulin and HOMA-IR^2^ did not reach statistical significance.

**Table 1:** Metabolic parameters of treated hamster groups.

	Chow diet	Chow diet + AG	Western diet	Western diet + AG
Body Weight (g)	141.6 ± 2.63	145.3 ± 4.19	142.07 ± 3.61	154.8 ± 2.71^£*^
Total Body weight gain (g)	30.6 ± 2.18	34.13 ± 4.00	30.6 ± 4.29	41 ± 3.39
Body weight gain after pump implantation (g)	4.8 ± 0.6	13.4 ± 1.1^¥*^	6.1 ± 0.9	16.2 ± 0.9^¥£^
Food intake (kcal/2 days)	86.70 ± 32.02	89.7 ± 30.89	50.36 ± 11.12	61.2 ± 12.69
Liver weight (g)	3.76 ± 0.24	3.77 ± 0.08	5.75 ± 0.28^¥£^	6.90 ± 0.23^¥£*^
Small intestine weight (g)	1.71 ± 0.04	1.65 ± 0.06	1.95 ± 0.10^¥£^	1.99 ± 0.04^¥£^
Colon weight (g)	1.03 ± 0.08	1.12 ± 0.09	0.66 ± 0.02^¥£^	0.74 ± 0.03^¥£^
Epididymal fat (g)	1.5 ± 0.13	1.34 ± 0.14	1.24 ± 0.17	2.02 ± 0.13^¥£*^
Blood glucose (mM)	4.9 ± 0.50	5.81 ± 0.63	6.28 ± 0.31	6.38 ± 0.60
Plasma insulin (pM)	60.48 ± 6.91	62.96 ± 9.46	82.88 ± 13.88	102.94 ± 14.33
HOMA-IR	2.45 ± 0.06	2.71 ± 0.58	3.53 ± 0.41	4.10 ± 0.61
Plasma TAG (mg/dL)	94.76 ± 18.17	111.03 ± 32.47	322.4 ± 52.09^¥£^	637.42 ± 48.09^¥£*^
Plasma total cholesterol (mg/dL)	71.93 ± 8.42	96.66 ± 4.28	212.21 ± 10.12^¥£^	255.35 ± 13.59^¥£*^
Plasma free cholesterol (mg/dL)	43.34 ± 6.80	43.7 ± 3.73	66.79 ± 2.81^¥£^	84.37 ± 4.09 ^¥£(p=0.05)^
Plasma cholesteryl ester (mg/dL)	38.60 ± 7.55	55.93 ± 1.81	145.41 ± 7.53^¥£^	168.52 ± 15.35^¥£^
Plasma HDL-C (mg/dL)	26.35 ± 5.02	20.57 ± 2.43	35.31 ± 3.46	47.30 ± 4.23^¥£^
Plasma non HDL-C (mg/dL)	54.37 ± 7.87	77.57 ± 3.77	176.89 ± 8.57^¥£^	203.38 ± 16.99^¥£^
Hepatic TAG (mg/g tissue)	0.013 ± 0.001	0.013 ± 0.001	0.024 ± 0.004^¥£^	0.020 ± 0.002
Hepatic cholesterol (mg /g tissue)	3.53 ± 0.16	3.90 ± 0.24	20.71 ± 4.77^¥£^	18.64 ± 2.49^¥£^
Small intestine TAG (mg/g tissue)	33.56 ± 5.61	16.63 ± 4.29^¥^	24.01 ± 1.92	20.39 ± 3.42
Small intestine cholesterol (mg/g tissue)	1.23 ± 0.24	2.72 ± 0.35^¥^	5.40 ± 0.11^¥£^	3.97 ± 0.29^¥£*^

Results are presented as mean ± SEM (n = 6–8); ^¥^P < 0.05 *vs* chow diet, ^£^P < 0.05 *vs* chow diet + AG; ^*^P < 0.05 *vs* Western diets.

### Acylated ghrelin and ERK1/ERK2 activation in the small intestine

**Figure 5:**
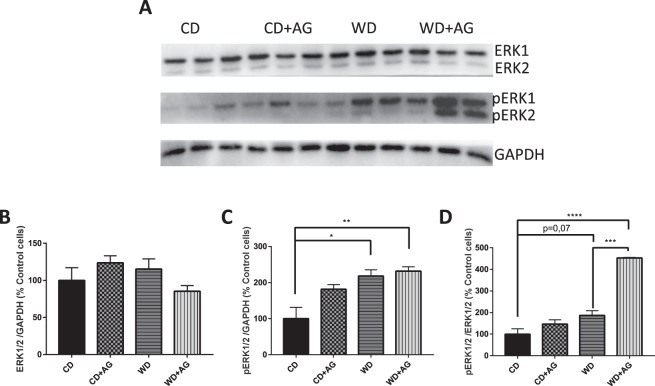
Effect of acylated ghrelin on ERK1/ERK2 activation in the jejunum. Syrian Golden Hamsters were submitted to conventional chow (CD) or Western (WD) diets for 8 weeks. For the last 2 weeks of treatment, animals were continuously administered with acylated ghrelin (AG, 100 nmol/kg/day) or physiological saline. Before sacrifice, hamsters were fasted for 16 h before receiving an olive oil gavage (200 µL). Jejunum samples were homogenized in a lysis buffer and proteins lysates were analyzed by Western Blot with ERK1/ERK2 (**A,B**) and phosphoERK1/ERK2 (**A,C**) antibodies as indicated. The ratio of pERK1/2/ERK1/2 was also analyzed. (**D**) For accurate normalization, the same blot was probed with GAPDH. A representative blot is shown, illustrating an experiment in triplicate on the same gel and at the same time exposure. The results of every experiment are shown as mean ± SEM of n = 4–6 animals ^*^P < 0.05; ^**^P < 0.01; ^***^P < 0.001; ^****^P < 0.0001.

To further characterize the increase in the weight of the small intestine, measurements of ERK1/ERK2 and phospho-ERK1/ERK2 levels (proteins known to regulate cell proliferation) were performed^21^. Whereas no significant difference was noted in ERK1/2 protein expression among animal groups (Fig. 5A,B), an increase was observed in phosphoERK1/ERK2 in response to WD feeding (Fig. 5A,C). The phosphorylation was accentuated for animals submitted to WD + AG (Fig. 5D)

### Acylated ghrelin and chylomicrons output in Syrian golden hamsters

**Figure 6:**
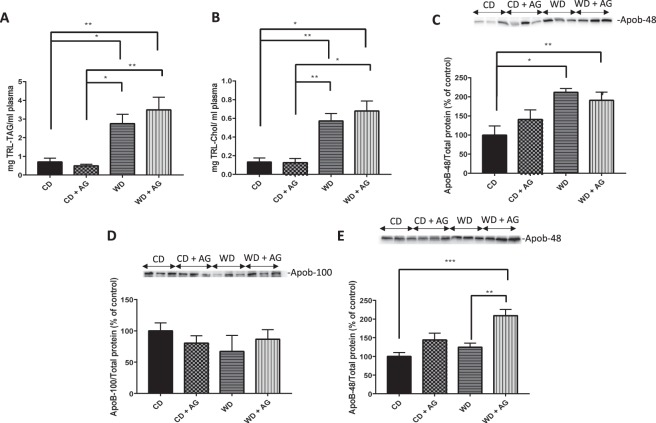
Effect of acylated ghrelin on chylomicron secretion output. Syrian Golden Hamsters were submitted to conventional chow (CD) or Western (WD) diets for 8 weeks. For the last 2 weeks of treatment, animals were continuously administered with acylated ghrelin (AG, 100 nmol/kg/day) or physiological saline. Before sacrifice, hamsters were fasted for 16 h, followed by an olive oil gavage (200 µL). After 20 minutes, they were peritoneally injected with 0.5 mg/kg of Pluronic F-127 to inhibit LPL-induced chylomicron (CM) catabolism. Two hours after gavage, hamsters were sacrificed by an intracardiac exsanguination. Blood was collected and processed to isolate and measure triglyceride-rich lipoproteins (TRL) content. Triacylglycerol (**A**) and total cholesterol (**B**) concentrations were determined. The apoB-48 content (**C**) was obtained by Western blots and results are illustrated as densitometric ratios of apoB-48 to protein content (stain-free gels). The apoB-100 (**D**) and apoB-48 (**E**) content of the fasted plasma taken by the tail was also analyzed by Western blots. A representative blot is shown, illustrating an experiment in triplicate on the same gel and at the same exposure. The results of every experiment are shown as mean ± SEM of n = 3–6 animals ^*^P < 0.05; ^**^P < 0.01; ^***^P < 0.001.

After a 16h-fasting, animals were given an olive oil gavage, and CM catabolism was inhibited by poloxamer 407. Two hours later, the triacylglycerol-rich lipoprotein (TRL) fraction containing CMs and VLDLs was isolated from the plasma. This fraction mostly contained CMs (in view of dietary lipids given to the animals), and to a lesser extent, VLDL particles (due to similarities in their respective density values: d < 1.006 g/L). To ascertain that the lipid contents are representative of intestinal TRL, the analysis of apoB-48 levels in the plasma was also included. Compared to the CD, the WD diet increased TAG (Fig. 6A) and cholesterol content (Fig. 6B) in the TRL fraction while also raising apoB-48 levels (Fig. 6C). On the other hand, AG had no effect on the plasma moieties of TRL, e.g. TAG (Fig. 6A), cholesterol (Fig. 6B) and apoB-48 (Fig. 6C), indicating that AG did not influence postprandial CM secretion in hamsters under our experimental conditions. In the fasted condition, WD had no significant effect on circulating apoB-100 (Fig. 6D) and apoB-48 (Figure E), but AG specifically raised apo B-48 levels in animals submitted to WD (Fig. 6E).

### Acylated ghrelin and lipid accumulation in the small intestine

**Figure 7:**
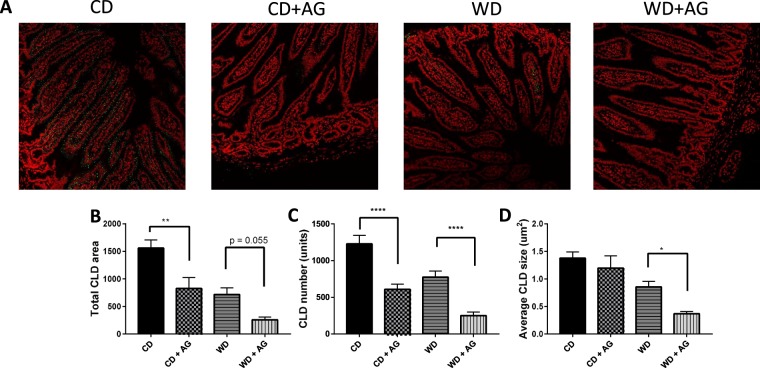
Effect of acylated ghrelin on CLD accumulation in the jejunum. Syrian Golden Hamsters were submitted to conventional chow (CD) or Western (WD) diets for 8 weeks. For the last 2 weeks of treatment, animals were continuously administered with acylated ghrelin (AG, 100 nmol/kg/day) or physiological saline. Before sacrifice, hamsters were fasted (16 h), received an olive oil gavage (200 µL) and after 20 minutes, they were peritoneally injected with 0.5 mg/kg of Pluronic F-127 to inhibit LPL-induced chylomicron catabolism. Two hours later, hamsters were sacrificed, sections of the jejunum were collected, fixed and prepared for staining with DAPI (red, nuclei) and LipidTox (green, neutral lipids). Representatives images for every treatment are shown (**A**) and cytosolic lipid droplet (CLD) total area (**B**), number (**C**) and average size (**D**) were characterized. Results are shown as mean ± SEM (n = 4–6); ^*^P < 0.05; ^**^P < 0.01; ^****^P < 0.0001.

Most of the lipids internalized by enterocytes are secreted as CM or accumulated in the cytosol as CLDs^7^. Since jejunum is one of the most critical site for lipid absorption in the intestine^22^, we investigated how the diets and AG influence its capacity to generate CLDs. A potent reduction of CLD total area and numbers was observed in the jejunum of WD hamsters (Fig. 7A–C). Hence, AG significantly decreased CLD total area and numbers both in WD and CD animals (Fig. 7A–C).

## Discussion

Cytosolic lipid droplets are important organelles containing a core of TAG and cholesteryl ester surrounded by a phospholipid monolayer^23^. They are found in many tissues where they are proposed to play distinct physiological roles. For instance, CLD hypertrophy is associated with energy storage and the progression of cardiometabolic disorders in adipose tissues and liver, leading to obesity and NAFLD, respectively^24,25^. The interest towards understanding the physiological relevance of CLDs in the intestine has grown over the recent years and the mechanisms underlying their production and synthesis are under tight regulatory control. Once lipolytic products are absorbed from the intestinal lumen, they are transported to the ER membrane for lipid re-esterification and are used for the synthesis and secretion of CM into the circulation. The lipolytic products may also serve for the CLD production^26^. As reviewed, only a few GI factors are known to modulate CLD synthesis and accumulation in the intestine^4^. One potential regulator of CLD production is AG, a GI peptide known to increase appetite while promoting lipid storage in adipose tissues and liver^27^. Surprisingly, the effect of AG on lipid metabolism in the intestine has been overlooked over the last years. The first objective of the present study was, therefore, to appraise the relation between CM production/secretion and CLD accumulation using cultured Caco-2/15 enterocytes and employing Syrian Golden Hamsters. The second objective was to identify how AG modulates lipid metabolism in the intestine. The major findings show that CLD growth was increased in response to prolonged exposure to high lipid concentrations and this effect was even higher in response to CM inhibition in Caco-2/15 enterocytes. In WD hamsters, CLD content was reduced while postprandial CM secretion was increased. Prolonged AG treatment amplified the dyslipidemia induced by WD, reduced CLD accumulation in the intestine and increased CM output. Overall, our data emphasize a strong connection between CM biogenesis and CLD accumulation.

The present results show that CLD production and hypertrophy were increased in response to prolonged exposure to higher concentrations of oleic acid (OA). The optimal conditions were met when Caco-2/15 cells were incubated with 600 μM of OA for 24 h, using two fluorescent markers for nuclei (DAPI) and neutral lipids/CDLs (LipidTox), confocal microscopy and image analysis. The latter method was instrumental in characterizing the effects of OA on CLD accumulation in Caco-2/15 cells. In fact, it allowed determining the outcomes of the different treatments on CLD total area and numbers per microscopy field. Characterization of CLDs was always performed using a monolayer of differentiated Caco-2/15 cells plated on transwell filters at 100% confluence. This was critical for standardizing the method. Confocal microscopy was previously proposed as one of the most efficient tools for characterizing CLDs^28^. Using a method similar to the one reported in the present study, OA (600 µM) was shown to increase CLD production by 100% in Caco-2 cells^29^. This effect is potentially more important in Caco-2 enterocytes since these cells do not secrete as much lipoproteins as primary enterocytes. Perilipin 2 (Plin-2) and intracellular TAG levels were also measured, which confirmed the results obtained with the fluorescent markers/confocal microscopy.

In our hands, Lomitapide Mesylate inhibited MTP activity and decreased CM production/secretion by 69%, which concomitantly increased total CLD area per microscopy field. Following their uptake by enterocytes of the jejunum, NEFAs are transported to the ER by both I-FABP and liver FABP (L-FABP)^30^. Once transported to the ER, NEFAs are re-esterified as TAG and enter the ER where CM or CLD synthesis is achieved^31^. CM synthesis requires TAG assembly with apoB-48 under the action of MTP^22^. This suggested that MTP inactivation or blockage could prevent CM synthesis and promote CLD production. The present results support the hypothesis that the inhibition of CM production/secretion increases CLD synthesis to prevent intracellular lipotoxicity in the intestine. Our data clearly evidence the intimate relationship between CLD accumulation and CM secretion.

There is little information about the effects of AG on intestinal functions. AG was previously reported to promote enterocyte proliferation in the intestine. However, the present study is the first to investigate its role in lipid transport in enterocytes^32^. According to our findings, AG down-regulated FA uptake in enterocytes. This effect was not initially expected since our group previously reported that AG potentiates NEFA uptake in differentiated 3T3-L1 cells^16^. However, the present data are supported by the observation that AG reduces palmitic acid transport in undifferentiated myoblasts^33^. This indicates that AG exerts tissue-specific actions on NEFA uptake. However, in the conditions tested, AG did not influence lipoprotein secretion or CLD accumulation in Caco-2/15 cells. Treatment with AG significantly reduced NEFA transport at 100 pM, a concentration that was previously shown to be “optimal” according to our group^16^. This treatment with 100 pM of AG can also be considered physiological since plasma concentrations of AG in humans range from 50–300 pM^34,35^. Several factors could have influenced the present results in Caco-2/15 cells. For instance, some peripheral outcomes of AG were reported to be mediated by factors related to the CNS^36^. Also, it is possible that AG increased the rate of CM production/secretion but this effect was no longer noticeable after 24 h of incubation. Previously, Caco-2/15 cells provided critical information on how enterocytes absorb lipids and release them as CMs. Although they were originally isolated from the colon, their capacity to metabolize lipids following full differentiation is closely related to the capacity of enterocytes in the small intestine^37^. However, they display a reduced capacity to secrete lipoproteins (approximately 15–20% of all TAG molecules) and are proposed to synthesize more CLDs than native enterocytes of the small intestine^38^. Based on these factors, it was critical to validate these results *in vivo*.

The Syrian Golden Hamster is considered as one of the best rodent models for human lipoprotein production in physiological and pathophysiological conditions. In contrast to mice and rats, hamsters also express cholesteryl ester transfer protein (CETP) responsible for TAG and cholesteryl ester exchange between VLDL/LDL and HDL particles^39^. Such a model was previously used to characterize the effects of GI peptides, e.g. glucagon-like peptide 1 (GLP-1) and 2 (GLP-2), on CM secretion^10,40^. When submitted to WD, hamsters developed obesity, dyslipidemia and severe hepatic steatosis, as well as inflammation and reduced weight of their colon^41^. In the present study, hamsters submitted to WD did not develop obesity when compared to CD animals. However, dyslipidemia was present along with elevated lipid content and weight in the small intestine and liver in response to WD. In contrast, reduced colon weight was observed in WD animals. Although not initially expected, resistance to diet-induced obesity and the absence of insulin resistance were previously reported in hamsters and rats by other groups^42–46^.

As mentioned above, the weight of the small intestine was higher in the WD group compared to control diet and was even more increased with AG administration. These results were surprising given the lack of differences in TAG content. To understand these observations, we examined whether other lipid species and cell proliferation may contribute to the weight increment of the small intestine. Indeed, cholesterol content and phosphorylated ERK expression (as a biomarker of cell proliferation) were both elevated, which may play a role in intestinal weight gain. Noteworthy, ERK is an important modulator of cell proliferation in different tissues such as the small intestine^47–50^ and AG was shown, in several studies, to positively influence cell proliferation in many tissues such as intestinal epithelial IEC-6 cells^51–54^.

In hamsters submitted to CD, continuous AG treatment significantly reduced intestinal CLD total area and numbers per microscopic field. However, AG did not induce any other change in the metabolic phenotype of CD hamsters. In response to WD, AG infusions significantly increased body weight, epidydimal fat and liver weight. Surprisingly, this effect was not associated with an increased caloric intakes, but AG was previously proposed to promote obesity through other mechanisms^55,56^. The most striking differences observed in hamsters submitted to WD and AG infusion were related to the development of dyslipidemia (increased fasting plasma TAG and cholesterol levels). Since AG was previously reported to promote hepatic steatosis^57^, liver TAG and cholesterol levels were measured. In contrast to what was observed in the mouse model in physiological and pathophysiological conditions, AG infusion did not increase hepatic TAG and cholesterol concentrations.

In WD hamsters, olive oil gavage promoted CM secretion, this was validated by an increase in TRL lipid contents and apoB-48 levels in plasma, while decreased CLD production in the jejunum was also observed. Our results confirm previous data showing that lipid-rich diets increase CM secretion through an adaptive mechanism intimately associated to insulin resistance^58,59^. In the present study, the higher weight of the small intestine observed in WD animals, suggests an adaptation of the small intestine to dietary fat and sucrose consumption, potentially increasing not only the absorptive surface of the small intestine but also its weight as pointed out above^60^.

In contrast, CD animals displayed reduced CM secretion and increased CLD production by the jejunum. This indicates that hamsters on CD accumulated TAG in CLDs rather than releasing them for the formation and secretion of CM particles. In the conditions tested, AG did not influence CM secretion but it significantly reduced CLD accumulation in CD and WD hamsters. These results are intriguing and indicate that, in response to AG, TAG could be hydrolyzed and utilized through other mechanisms than those involved in lipoprotein production. For instance, AG could prevent TAG re-esterification and direct NEFAs towards β-oxidation^61^ rather than CLD or CM production. Since AG reduces lipid absorption in cultured enterocytes, its obesogenic effects could be mediated via mechanisms which bypass intra-enterocyte lipid uptake and involve increased intestinal permeability. Approximately 40% of lipids are directly transported via the portal circulation instead of being re-esterified and sequestered in the ER for CM production/secretion^62^. However, the mechanisms underlying these effects are still unknown. If proven right, AG could potentiate fat absorption through the portal circulation, which promptly stimulates lipid uptake in the liver and adipose tissues^16,57^. This effect could also be explained by the negative correlation between the concentration of ghrelin and certain bile acids^63^. If confirmed, AG could reduce lipid emulsification and absorption in the intestine. Although it is not supported by the present *in vitro* data, it is also possible that AG promptly enhanced CM secretion while this effect was no longer detectable 2 h after the olive oil gavage. Finally, AG could also influence other yet undefined mechanisms regulating lipid metabolism in the intestine. It is therefore critical to conduct further investigations on the role of AG in lipid metabolism in the intestine. Importantly, we cannot exclude the possibilities that, under the influence of a Westernized type of diet, lipoprotein lipase could be altered.

The high levels of plasma TAG and cholesterol in fasting conditions indicate the presence of dyslipidemia following AG administration. As the liver is usually a significant contributor of VLDL, it was important to determine apoB-100, the most characteristic protein of VLDL. Our findings stress no increment in apoB-100, which suggests the lack of VLDL overproduction by the liver, thereby ascribing the dyslipidemia to intestinal CM output. An explanation for the failure of the liver to enhance VLDL secretion in response to WD and WD + AG may be associated with the extremely high level of cholesterol crystals, the macro and micro steatosis, and the reduction of hepatocytes size. Additional efforts are needed to delineate the mechanisms.

In conclusion, the present results indicate that AG plays a role in fat absorption, uptake or metabolism through yet undefined mechanisms in the intestine. This report is first to show that in addition to its adipogenic effects, AG also promotes dyslipidemia via intestinal lipoprotein secretion under the fasted state. This suggests the critical need to use an integrative approach to determine how GI peptides regulate lipid metabolism in key metabolic organs such as the intestine.

## Materials and Methods

### Enterocyte culture

Caco-2/15 cells were cultured at 37 °C and 5% of CO_2_ in EMEM medium (Wisent Inc., Canada) containing 10% fetal bovine serum (FBS; Wisent Inc.), 1% penicillin/streptomycin and 1% non-essential amino acids (GIBCO, USA) as described previously^64,65^. Cells were maintained in T-75 cm^2^ flasks (Corning Inc., USA) until they reached 80–90% of confluence and were trypsinized. For individual experiments, enterocytes were seeded at the density of 1 × 10^6^ cells/well on polycarbonate 24.5-mm transwell filters with pores of a diameter of 0.4 µm (Corning Inc., USA). Differentiation occurred when enterocytes were cultured for 15 days post-100% confluence.

### *In vitro* lipoprotein output and isolation by ultracentrifugation

Radiolabeled [^14^C]-oleic acid (Perkin Elmer, USA) was added to unlabelled oleic acid and both were conjugated to fatty acid-free bovine serum albumin (BSA). This preparation (600 µM of unlabelled + 0,45 µCi of radiolabeled oleic acid) was added to the apical side of differentiated Caco-2/15 cells for 24 h. For investigating the role of ghrelin on lipid metabolism, enterocytes were also incubated 24 h with AG (100 pM) on basolateral and apical sides with or without 50 µM of a GHSR-1a antagonist [D-Lys^3^]-GHRP-6 (Tocris Bioscience, UK). Following the incubation period, enterocytes were scraped in cold PBS containing 1 mM of pepstatin A, PMSF, and aprotinin. The basolateral medium was collected and lipid-rich plasma (as a carrier for the lipoproteins synthesized by Caco-2/15 cells) was added (4:1 ratio). The lipoproteins were isolated by ultracentrifugation (OptimaMax Ultracentrifuge, Beckman Coulter, USA): chylomicrons (CM) at 25,000 rpm for 40 min, VLDL and LDL at densities of 1.006 g/mL and 1.063 g/mL, respectively, at 100,000 rpm for 2h30, HDL at density of 1.21 g/mL and at 100,000 rpm for 6 h. Scintillation liquid (Ecolite, MP Biomedicals, USA) was added to the isolated fractions in order to determine the radioactivity derived from the labeled [C^14^]-oleic acid using a β counter (Hidex 300, Finland). To evaluate the impact of CM secretion on cytosolic lipid droplets, microsomal triglyceride transfer protein (MTP) was inhibited for 24 h by the addition of 0.1 µM Lomitapide Mesylate (Selleck chemicals, USA) on the apical compartment in presence of OA.

### Cytosolic lipid droplet analysis and characterization

For CLD characterization, Caco-2/15 cells were incubated 24 h in serum-free EMEM medium using Transwell filters. Cells were then incubated with BSA-conjugated OA (0, 75, 150, 300 and 600 µM) for 1, 6, 12 and 24 h. After the incubation, nuclei were stained with 1.5 ng/ml 4′,6-diamidino-2-phenylindole (DAPI, Molecular Probes, USA) in PBS for 5 minutes and washed again in PBS. Finally, their CLDs were stained for 30 minutes with LipidTox (ThermoFisher Scientific, Canada) diluted 1/1000 in PBS and mounted using 50% glycerol/PBS. Fluorescence was observed with an inverted TI microscope and imaged with an A1 confocal unit (Nikon, Japan). The total area and numbers of cytosolic lipid droplets per microscopic field were obtained with the Image J Software (NIH, USA).

### Role of acylated ghrelin in fatty acid uptake

Differentiated Caco-2/15 cells were preincubated in serum-free EMEM medium for 2 h before being treated with AG (10 pM, 100 pM, 1 nM, 10 nM) in PBS. FA uptake was then assessed using a commercial kit (QBT fluorescence FA assay kit, Molecular Devices, USA). Briefly, 100 µL of BODIPY reagent (diluted in Hank’s balanced salt solution to 0,2%) was added to Caco-2/15 enterocytes. Intracellular FA uptake was evaluated continuously with λ_ex_ = 485 nm and λ_em_ = 515 nm every 30 sec for 2 h with a Spectramax i3x (Molecular Devices, USA), according to the manufacturer’s instructions.

### Effects of acylated ghrelin on lipid esterification

Caco-2/15 cells were incubated with AG as described above. Cellular and basolateral medium lipids were extracted overnight in chloroform-methanol (2:1). Lipids were then separated by thin layer chromatography (TLC, Millipore Sigma, USA) and their corresponding bands were scratched off the plates and disposed in scintillation vials containing EcoLite counting fluid (MP BIOMEDICALS, LLC). Radioactivity was measured by scintillation counting (Hidex 300, Finland).

### Syrian Golden Hamsters and lipid metabolism in the intestine

All animal protocols were approved by UQAM’s Institutional Animal Care Committee and were performed in accordance with the relevant guidelines and regulations of the institution (CIPA: #0515-R3-759-0519). Nine-week old (100–110 g) male Syrian Golden Hamsters (Envigo, USA) were acclimatized for 1 week under controlled environmental conditions (fed *ad libitum* at 22 °C with 12 h light/dark cycles). Thereafter, hamsters were submitted to conventional chow (CD, Rodent Chow 5075, Charles River, Canada) or Western (WD, #D12492, Research Diet Inc. USA) diets for 8 weeks. Body weight and food intake were measured every second day. Six weeks after initiating WD or CD, animals were anesthetized with 3.5–4.5% isoflurane_._ A small intrascapular incision was performed under aseptic conditions to insert an osmotic minipump (ALZET model 2002, DURECT Corporation, USA) containing AG (100 nmol/kg/day; Polypeptide, France) or physiological saline. The solutions were administered subcutaneously for 14 days. The selected concentration of AG was previously shown to increase food intake in rats^66^. After the implantation of mini-pumps, hamsters were injected with a subcutaneous dose (5 mg/kg) of Ketoprofen (Merial, Canada).

### Liver histology and electronic microscopy

For hematoxylin-eosin staining, tissues were fixed in formalin 10% and embedded in paraffin. Thereafter, they were processes for light microscopy with hematoxylin-eosin. Images of stained tissues were captured by a Zeiss Imager A1. Measurements were taken with the axiovision software. For electronic microscopy, the liver was cut into small pieces and fixed with 3% glutaraldehyde. Afterwards, it was post-fixed with 1% osmium tetroxide, followed by a consecutive incubation in an ascending acetone series (50%, 70%, and 90%), with a final incubation in 100% ethanol for hydration. After incubation, the tissues were embedded in Epon. 1-nm sections were cut and stained using uranyl acetate and lead citrate. Electron microscopy images were taken with the Phillips EM208 electron microscope.

### Estimation of *in vivo* postprandial chylomicron production

Two weeks after mini-pumps were inserted, hamsters were fasted for 16 h and 500 µL of blood was collected from the tail vein under anesthesia with isoflurane/O_2_, before receiving an oral gavage of pure olive oil (200 µL). Twenty minutes after gavage, animals were injected with an intraperitoneal (ip) dose (0.5 mg/kg) of Pluronic F-127 (poloxamer 407; 10% in saline, Millipore Sigma, USA) to inhibit lipoprotein lipase activity and prevent CM catabolism. Two hours after gavage, animals were sacrificed by cardiac exsanguination and blood was transferred into EDTA-containing tubes (Vacutainers, B&D, USA). Blood was centrifuged at 6000 rpm for 10 minutes at 4 °C. Plasma and isolated tissues were flash-frozen and kept at -80 °C for further analyses. Fresh plasma was used to isolate triglyceride-rich lipoproteins (CM and VLDL) by ultracentrifugation. Briefly, 250 µL of plasma was added to 4 mL of a sodium chloride buffer (density of 1.006 g/L) and centrifuged at 35,000 rpm for 70 minutes at 4 °C. The upper fraction was removed and its lipid moieties, TAG and cholesterol, were respectively determined by commercial kits from Randox Laboratories Ltd (United Kingdom) and Wako Diagnostics (USA).

### Biochemical analyses in fasting plasma

Fasting TAG, HDL-cholesterol (Bio Scientific, USA), insulin (Mercodia, Sweden), total cholesterol and free cholesterol were analyzed with commercial kits (Wako Diagnostics, USA). Fasting glucose was measured with a glucometer (Contour^®^ NEXT, Bayer, Germany) and HOMA-IR was used as an insulin sensitivity index as previously reported^67^.

### CLD characterization using fluorescent markers and confocal microscopy

Hamster jejunum tissues were incubated in 4% paraformaldehyde (PFA) and then embedded in O.C.T (Fisher Healthcare, USA). Caco-2/15 treated as mentioned above were washed with PBS and fixed with PFA 4% for 15 min. Staining of cryosections were performed as described in *Section* 2.2.

### Lipid analysis in the jejunum

Approximately 0.1 g of jejunum tissue or liver was homogenized in an EDTA buffer and lipids were extracted in a 2: 1 chloroform/methanol solution for 18 h. After evaporating the inferior phase with N_2_ gas, lipids were resuspended in 800 µL of nanopure H_2_O (Millipore, USA). TAG and total cholesterol were analyzed with the same kits used for the biochemical analysis. For phospholipids quantification, tissue homogenates were analyzed using the Bartlett method^68^. In short, homogenates were diluted in perchloric acid and the solution was heated at 160 °C for 3 hours. Water, ammonium molybdate, and 2.4-Diaminophenol Dihydrochloride were incorporated and heated another 7 minutes at 96 °C. Phospholipid concentrations were determined at an absorbance of 830 nm.

### Western blot analysis

For Western Blots, 1 µL of plasma or 20 µg of proteins was heated for 10 min at 80 °C with a buffer containing sodium dodecyl sulfate (SDS) and dithiothreitol (DTT). Samples were loaded and migration was achieved on 6% SDS-PAGE gels while protein transfer was performed on a nitrocellulose membrane (GE Healthcare Lifescience, Canada). The membrane was then blocked for 1 h in TBS-Tween buffer containing 5% of skimmed milk and washed in TBS-Tween buffer. Incubation was then achieved overnight with the appropriate primary antibody. The antibodies used are: apoB-48 primary antibody at 1/1000 in milk (apoB, MAB012, Millipore Sigma, USA), Plin-2 primary antibody at 1/250 in milk (Plin-2, 690102, Progen, Germany), ERK1/ERK2 primary antibody at 1/1000 in milk (ERK1/ERK2, 61–7400, ThermoFisher, Canada) and phospho-ERK1/ERK2 (phospho-ERK1/ERK2, MA5-15173, ThermoFisher, Canada). A specific secondary antibody conjugated with horseradish peroxidase was used to detect the relative amount of primary antibody. Bands were analyzed with the ImageLab software (Biorad). For apoB-48, total protein content was determined using stain-free gels (Bio-Rad, USA) and sample normalization was carried out using these values, as previously described^69^. For Plin-2, β-actin (1:250 000, Sigma-Aldrich, USA) was used for sample normalization while GADPH (1:1000, Abcam, USA) was used for ERK and phosphoERK.

### Statistical analysis

Results are presented as mean ± SEM. Statistical analyses were conducted using ANOVA and the differences between means were analyzed using *post-hoc* Turkey tests. If the experiment did not pass the Shapiro-Walk normality test, the Kruskal-Wallis test was used. Significance is considered at P < 0.05.

## Data Availability

The datasets generated during and/or analysed during the current study are available from the corresponding author on reasonable request.
